# Callispheres® drug-eluting beads transarterial chemoembolization might be an efficient and safety down-staging therapy in unresectable liver cancer patients

**DOI:** 10.1186/s12957-022-02717-9

**Published:** 2022-08-09

**Authors:** Ning Peng, Linfeng Mao, Yiwen Tao, Kaiyin Xiao, Guandou Yuan, Songqing He

**Affiliations:** 1 Department of Hepatobiliary Surgery, The First Affiliated Hospital of Guangxi Medical University, Nanning, 530021 Guangxi China; 2 Key Laboratory of Early Prevention and Treatment for Regional High Frequency Tumor, Guangxi Medical University, Ministry of Education, Nanning, 530021 Guangxi China; 3 Guangxi Key Laboratory of High-Incidence-Tumor Prevention & Treatment, Guangxi Medical University, Nanning, 530021 Guangxi China; 4 Department of General Surgery, Guangxi International Zhuang Medicine Hospital, Nanning, 530000 Guangxi China

**Keywords:** Callispheres® drug-eluting beads transarterial chemoembolization, Down-staging, Unresectable liver cancer patients, Tumor diameter, Residual liver volume

## Abstract

**Purpose:**

The purpose was to explore the effect of drug-eluting beads transarterial chemoembolization (DEB-TACE) on down-staging in unresectable liver cancer patients.

**Methods:**

A total of 180 patients with PHC treated by TACE were retrospectively analyzed. These included 80 cases in the DEB-TACE group and 100 cases in the cTACE group. Of these, 56 had complete clinical data (DEB-TACE: 24, cTACE: 32), and 23 patients received hepatectomy after TACE as a down-staging therapy (DEB-TACE: 15, cTACE: 8). Data (including clinical characteristics, clinical efficacy, tumor response, tumor diameters, residual liver volume, and liver function indexes before and after TACE, RFS, OS, and complications were collected and compared. Treatment response was evaluated at 1 month after TACE. Tumor diameter was evaluated by abdominal computed tomography scan. The residual liver volume was evaluated by IQQA liver system, and relapse-free survival (RFS) and overall survival (OS) were calculated by Kaplan–Meier curves.

**Results:**

The conversion rate in DEB-TACE group was higher than cTACE group (18.8% vs 8%, *p* = 0.032). In DEB-TACE group, 17 patients achieved objective response rate (ORR) which was higher than cTACE group (70.8% vs 34.4%, *p* = 0.007). The tumor necrosis rate was higher in DEB-TACE group, but there was no significant difference between the two groups (*p* = 0.053). Tumor diameter was decreased after TACE compared to before TACE (DEB-TACE: 9.4 ± 3.3 vs. 5.4 ± 3.5 cm, *p* = 0.003; cTACE: 9.7 ± 2.6 vs. 6.9 ± 2.2, *p* = 0.036). As to residual liver volume, it was increased after TACE compared to before TACE (1066.2 cm^3^ vs. 1180.3 cm^3^, *p* = 0.007) in DEB-TACE group, while there was no significant difference in cTACE group (1046.4 cm^3^ vs. 1170 cm^3^, *p* = 0.339) compared by paired-sample *t*-test, but there was no significant difference before and after TACE when compared by unpaired-sample *t*-test (*p* > 0.05). After TACE at 1 month, the AFP level in the DEB-TACE group was significantly lower than that in the cTACE group (*p* = 0.003). For survival, the median RFS was 26.0 months in DEB-TACE group and 15 months in cTACE group; there was significant difference between the two groups (*p* = 0.0465). As to OS, the median OS in DEB-TACE group was higher than that in cTACE group, but there was no significant difference between the two groups (*p* = 0.165). For safety profiles, in terms of liver function and adverse events, there was no significant difference between the two groups.

**Conclusion:**

Compared with cTACE, DEB-TACE might be a more efficient and safety down-staging treatment in unresectable liver cancer patients.

## Introduction

Liver cancer is a kind of complex disease frequently arising in the setting of chronic liver disease and cirrhosis, which is considered as the sixth common diagnosed cancer and the second leading cause of death from cancer around the world, resulting in 841,080 new cases and 781,631 deaths during 2018 globally [1]. Despite the widespread use of surveillance programs (such as hepatic resection, liver transplantation, and image-guided tumor ablation), not all liver cancer patients could get benefits. For instance, hepatic resection is the optimally curable choice for liver cancer patients, particular in these patients at early stage, whereas most patients diagnosed as liver cancer are at intermediate or advanced stages and lost the best time to receive surgery due to unbearable invasion [2, 3]. Although liver transplantation is another curable treatment for patients with small multinodular tumors or advanced liver dysfunction, it is still limitedly applicated owing to the shortage of donators and strict blood type matching requirements [2]. In regard to image-guided tumor ablation, its most procedures are done percutaneously, and it could achieve complete necrosis of almost 100% in liver tumors smaller than 2 cm, while less effectiveness occurs in larger tumors [2, 4, 5].

For patients with unresectable liver cancer clinically, transarterial chemoembolization (TACE) is widely regarded as first-line therapy for the sake of reduced collateral damage to tumor-free parenchyma and increased treatment effect, which is divided into two types according to different chemotherapy modalities (including conventional TACE (cTACE) and drug-eluting beads TACE (DEB-TACE) [6, 7]. In detail, cTACE is a kind of conventional technology using Lipiodol as chemotherapy drug carriers, while it has been determined to have high systemic toxicity [8]. So as to solve this problem, DEB-TACE has been introduced, which is a novel drug-delivery embolization system using microspheres as embolic agents and loading with chemotherapeutic drugs to gradually release them into the target tumor. Despite the benefits of DEB-TACE with accurate drug delivery and permanent vascular embolization, it is still not a curative treatment, and its disadvantages also contain liver function deterioration and incomplete tumor necrosis [9]. Therefore, convincing treatments combining with the advantages of DEB-TACE and other curable therapy is needed to be explored. Taken together with the above mentioned, we hypothesized DEB-TACE has down-staging effects for unresectable liver cancer patients, and after down-staging, these patients could receive curable hepatic resection. However, little is known about the down-staging benefit in unresectable liver cancer patients. In an attempt to address this dilemma, we carried out this study with the purpose of investigating the effect of DEB-TACE on down-staging in unresectable liver cancer patients.

## Methods

### Patient’s selection

From May 2016 to June 2018, a total of 180 patients with PHC treated by TACE in The First Affiliated Hospital of Guangxi Medical University were retrospectively analyzed. These included 80 cases in the DEB-TACE group and 100 cases in the cTACE group. Of these, 56 had complete clinical data (DEB-TACE: 24, cTACE: 32), and 23 patients received hepatectomy after TACE; as a down-staging therapy (DEB-TACE: 15, cTACE: 8) were enrolled in this study, all of them were phone-called each month and outpatient followed up at regular intervals until October 2021. The screening criteria were as follows:

(i) Patients with clinical diagnosis of primary hepatocellular carcinoma (PHC) in accordance with the Guidelines for Diagnosis and Treatment of Primary Liver Cancer in China (2017 Edition)
(ii) Primary interventional treatment and had no history of treatment for liver cancer before TACE
(iii) BCLC stages B or C and CNLC stages IIb–IIIb
(iv) BCLC stage A or CNLC stage Ib–IIIa who presenting with large and multiple tumors led to liver involvement > 70% or liver involvement > 50% coexisting with severe cirrhosis and insufficient residual liver
(v) Child-Pugh grades A or B
(vi) Eastern Cooperative Oncology Group performance status (ECOG PS) 0–2
(vii) Presenting with poor liver function and potential risk of severe post-excision cirrhosis, incomplete compensation of residual liver function, and post-excision liver failure and coronary heart disease or other severe complications resulting in a higher surgical risk
(viii) Clinical data and follow-up documents were complete.

The exclusion criteria were as follows: (i) malignant tumor of other sites, (ii) other treatment or antineoplastic drugs were performed for the corresponding period, and (iii) unstable systemic disease or uncontrolled infection, (iv) not complicated with other malignancies. We had access to information that could identify individual participants during or after data collection. The detailed information about clinical characteristics of patients was shown in Table 1. The approval for this study was acquired from the Medical Ethics Committee of The First Affiliated Hospital of Guangxi Medical University, and the written informed consent or verbal agreement with tape recording was obtained from enrolled patients or their guardians.

**Table 1:** Comparison of clinical characteristics of patients between DEB-TACE and cTACE

Items	DEB-TACE (*N* = 80)	cTACE (*N* = 100)	*p*-value
Age (years), mean ± SD	50.8 ± 7.4	51.5 ± 6.8	0.510
Gender, no. (%)			
Male	63 (78.6%)	78 (78%)	0.903
Female	17 (21.4%)	22 (22%)	
Etiology, no. (%)			
HBV	53 (66.3%)	70 (70%)	0.897
HCV	10 (12.5%)	12 (12%)	
Alcohol	8 (10%)	10 (10%)	
Others	9 (11.2%)	8 (8%)	
Number of tumors, no. (%)			
1–3	65 (81.2%)	83 (83%)	0.760
≥ 4	15 (18.8%)	17 (17%)	
Largest tumor diameter (cm), mean ± SD	8.69 ± 0.44	8.35 ± 0.45	0.605
Portal vein invasion, no. (%)	13 (16.25%)	18 (18%)	0.757
Lymphadenectasis, no. (%)	15 (18.8%)	20 (20%)	0.833
Distant metastasis, no. (%)	8 (10%)	12 (12%)	0.671
Liver involvement, no. (%)			
≤ 50%	7 (8.7%)	15 (15%)	0.419
50–70%	60 (75%)	68 (68%)	
≥ 70%	13 (16.3%)	17 (17%)	
ECOG score, no. (%)			
0–1	73 (91.3%)	85 (85%)	0.203
2	7 (8.7%)	15 (15%)	
BCLC stage, no. (%)			
A	23 (28.7%)	27 (27%)	0.684
B	45 (56.3%)	53 (53%)	
C	12 (15%)	20 (20%)	
Child-Pugh stage, no. (%)			
A	70 (87.5%)	83 (83%)	0.401
B	10 (12.5%)	17 (17%)	
Cycles of DEB-TACE, no. (%)			
1–2	54 (67.5%)	76 (76%)	0.816
≥ 3	26 (32.5%)	34 (34%)	
Conversion rate, no. (%)			
Yes	15 (18.8%)	8 (8%)	0.032*
No	65 (81.2%)	92 (92%)	

*SD* standard deviation, *HB* hepatitis B, *HCC* hepatocellular carcinoma, *ECOG* Eastern Cooperative Oncology Group, *BCLC* Barcelona clinic liver cancer, *DEB-TACE* drug-eluting bead transarterial chemoembolization

*p < 0.05

### Baseline data collection

Data of enrolled patients were collected from medical documents, including (1) demographics (age and gender); (2) histories (drink, hepatitis B (HB), hepatic encephalopathy, hypertension, heart disease, and liver cirrhosis); (3) clinical features at diagnosis (number of tumors, largest tumor size, portal vein invasion, lymphadenectasis, distant metastasis, liver involvement, Eastern Cooperative Oncology Group (ECOG) score, Barcelona clinic liver cancer (BCLC) stage, and Child-Pugh stage); (4) cycles of DEB-TACE before hepatectomy; and (5) tumor response, tumor diameters, residual liver volume, and liver function indexes before and after TACE, RFS, OS, and complications.

### Treatment process

The CalliSpheres® microspheres (CSM) (Jiangsu Hengrui Medicine Co. Ltd., Jiangsu, China) were used as drug-eluting beads in the present study, which had the diameter ranging from 100 to 300 μm. Before operation, the CSM was loaded with pirarubicin (20~40 mg), which was prepared as previously described [10]. The TACE operation was conducted in the digital subtraction angiography (DSA) room. Briefly, the tumor-supplying vessels were identified by the hepatic angiography using segment or subsegment superselective catheterization, and the femoral artery was punctured using microcatheter, which were performed in accordance with previous study [11]. When the microcatheter was precisely inserted into the tumor-supplying vessel, in DEB-TACE group, 50 mg lobaplatin, 500 mg fluorouracil, and 10 mL Lipiodol were successively infused into the tumor-supplying vessel, and then, a bottle of CSM loading with pirarubicin were infused into the tumor-supplying vessel. For patients with huge tumor, 1~3 bottle(s) of blank CSM (not loaded with drugs) were added additionally for embolization following the infusion of CSM loading with pirarubicin. Finally, a spot of lipiodol was injected into the tumor-supplying vessel for preventing reverse flow of microspheres, resulting in a “sandwich” effect. Besides, pre-procedure and post-procedure treatments were carried out in line with the previous study [11]. In cTACE group, 50 mg lobaplatin, 500 mg fluorouracil, and 10 mL Lipiodol were slowly injected into the tumor-feeding artery through a microcatheter under fluoroscopic monitoring to avoid reflux of Lipiodol emulsion followed by the infusion of a gelatin sponge. The cTACE procedure was terminated when target blood flow interruption or tumor stain disappearance was observed. The dosage of chemotherapeutic drugs was adjusted according to the patient’s liver function tests. The amount of Lipiodol and chemotherapeutic drug emulsions was given according to the tumor size and tumor-feeding arterial blood flow as previously described [12]. With respect to the patients presenting limited efficacy by one cycle of TACE, if necessary, repeated TACE was administered for them. Finally, all patients underwent curative resection or palliative resection, which was depended on the efficacy of down-staging treatment by TACE.

### Assessments

The sum of the largest diameters of target tumors in arterial enhancement on computed tomography (CT), the residual liver volume, the BCLC stage, the Child-Pugh stage, and the tumor markers (alpha-fetoprotein (AFP), the liver indexes (albumin (ALB), total protein (TP), total bilirubin (TBIL), total bile acid (TBA), alanine aminotransferase (ALT), aspartate aminotransferase (AST)) were examined and documented before and after down-staging treatment by DEB-TACE. The response to DEB-TACE therapy was evaluated at 1 month after DEB-TACE by enhanced CT or magnetic resonance imaging (MRI) examination, and the response criteria were in line with the modified response evaluation criteria in solid tumors (mRECIST), as follows: (1) complete response (CR), disappearance of any intratumoral arterial enhancement in all target lesions; (2) partial response (PR), at least a 30% decrease in the sum of diameters of viable (enhancement in the arterial phase) target lesions; (3) stable disease (SD), any cases that did not qualify either PR or progressive disease (PD); and (4) PD, an increase of at least 20% in the sum of the diameters of the viable (enhancing) target lesions. Furthermore, objective response rate (ORR) was defined as CR + PR. In addition, the patients’ surgical type (curative resection or palliative surgery) was also recorded, and the adverse events occurred post DEB-TACE therapy were documented as well.

### Follow-up

After surgery, patients were regularly followed up by clinic visits or telephone calls. The last follow-up date was 09 September 2021. Relapse-free survival (RFS) was calculated from the date of surgical resection to the date of disease relapse or death, whichever occurred first. Overall survival (OS) was calculated from the date of TACE therapy to the date of death or last follow-up.

### Statistical analysis

The residual liver volume was evaluated by IQQA liver system (EDDA Technology, Inc., New Jersey, USA). Statistical data processing was conducted on SPSS 24.0 software (SPSS Inc, Chicago, USA), and the figure construction was performed on GraphPad Prism 6 software (GraphPad software Inc., San Diego, USA). Continuous data were described as mean and standard deviation (SD) or median and interquartile range (IQR); categorical data were displayed as number (percentage). Comparison of the sum of target tumor largest diameters and residual liver volume before and after down-staging treatment by TACE was determined by paired-sample *t*-test and unpaired-sample *t*-test. Numerical differences between groups were assessed by chi-square test for categorical variables and *t*-test for continuous variables. RFS and OS were illustrated using Kaplan–Meier curves and log-rank (Mantel-Cox) test. *p*-value < 0.05 was considered significant.

## Results

### Patients’ characteristics

A total of 180 patients with PHC treated by TACE were retrospectively analyzed. These included 80 cases in the DEB-TACE group and 100 cases in the cTACE group; the detail clinical characteristics of patients were shown in Table 1. For clinical features, in terms of age, gender, etiology, tumor number, largest tumor diameter, portal vein invasion, lymphadenectasis, distant metastasis, liver involvement, ECOG score, BCLC stage, Child-Pugh stage, and cycles of DEB-TACE, there was no significant difference between the DEB-TACE and cTACE group (all *p* > 0.05, Table 1). While in terms of conversion success rate which means that patients with unresectable liver cancer can obtain opportunity for surgery after TACE treatment, the conversion rate in DEB-TACE group was higher than cTACE group (18.8% vs 8%, *p* = 0.032, Table 1).

### Treatment response after DEB-TACE and cTACE

Many patients were lost to follow-up due to abandonment of treatment or referrals after one TACE treatment. Therefore, only 56 had complete clinical data (DEB-TACE: 24, cTACE: 32). After DEB-TACE treatment at 1 month, of these patients, in terms of CR, PR, SD, and PD, there was no significant difference between the DEB-TACE and cTACE group (all *p* > 0.05, Table 2). The ORR rate in DEB-TACE group was higher than that in cTACE group (*p* = 0.007, Table 2). As for tumors necrosis rate, although the tumors necrosis rate of the DEB-TACE group appeared to be slightly higher than that in cTACE group, this difference was not significant (*p* = 0.053, Table 2).

**Table 2:** Tumor response at 1 month between DEB-TACE and cTACE

Tumor response	DEB-TACE (*N* = 24)	cTACE (*N* = 32)	*p*-value
Tumor response (all)			
CR	5	2	0.125
PR	12	9	0.094
SD	5	13	0.117
PD	2	8	0.162
ORR	17	11	0.007*
Necrosis rate (ORR)			
30–50%	4	7	0.053
≥ 50%	13	4	

*p < 0.05

### Comparison of tumor diameters and residual liver volume before and after DEB-TACE and cTACE

Twenty-three patients received hepatectomy after TACE as a down-staging therapy (DEB-TACE: 15, cTACE: 8); we calculated the tumor diameters and the residual liver volume before and after DEB-TACE in these patients. Tumor diameter was decreased after TACE compared to before TACE (DEB-TACE: 9.4 ± 3.3 vs. 5.4 ± 3.5 cm, *p* = 0.003; cTACE: 9.7 ± 2.6 vs. 6.9 ± 2.2, *p* = 0.036). However, there was no significant difference in the residual liver volume changes before and after TACE between the DEB-TACE and cTACE group (all *p* > 0.05, Table 3) when comparison was determined by unpaired-sample *t*-test. As to residual liver volume, it was increased after TACE compared to before TACE (1066.2 cm^3^ vs. 1180.3 cm^3^, *p* = 0.007, Table 4) in DEB-TACE group, while there was no significant difference in cTACE group (1046.4 cm^3^ vs. 1170 cm^3^, *p* = 0.339, Table 4) compared by paired-sample *t*-test, but there was no significant difference before and after TACE when compared by unpaired-sample *t*-test (*p* > 0.05, Tables 3 and 4).

**Table 3:** Tumor diameters and the residual liver volume between DEB-TACE and cTACE

Parameter	DEB-TACE (*N* = 15)	cTACE (*N* = 8)	*p*-value
Tumor diameters (cm)			
Before treatment	9.4 ± 3.3	9.7 ± 2.6	0.826
After treatment Residual liver volume	5.4 ± 3.5	6.9 ± 2.2	0.209
Before treatment	1066.2 ± 294.2	1046.4 ± 159.2	0.862
After treatment	1180.3 ± 319.4	1074.0 ± 137.8	0.383

Comparison was determined by unpaired-sample *t*-test

**Table 4:** The residual liver volume before and after DEB-TACE and cTACE

No.	DEB-TACE group (*N* = 15)				cTACE group (*N* = 8)			
	Before DEB-TACE	After DEB-TACE	Increase rate (%)	p-value	Before cTACE	After cTACE	Increase rate (%)	*p*-value
1	1182.0	1064.0	−10.0		1157	1201	3.8	
2	1006.0	1083.0	7.7		935	1112	18.9	
3	1266.0	1407.0	11.1		1104	1121	1.5	
4	1021.8	972.8	−4.8	0.007	1211	1115	−7.9	0.339
5	705.0	832.0	18.0		854	864	1.2	
6	1514.0	1659.0	9.6		1125	1114	−0.97	
7	796.8	706.0	−11.4		801	856	6.8	
8	784.0	1169.0	49.1		1184	1209	2.1	
9	1547.0	1740.0	12.5					
10	1266.0	1358.0	7.3					
11	1371.0	1371.0	0.0					
12	1127.0	1347.0	19.5					
13	944.0	1066.0	12.9					
14	909.0	1100.0	21.0					
15	554.0	713.0	28.7					
Mean	1066.2	1180.3	11.4		1046.4	1074.0	2.6	

Comparison was determined by paired-sample *t*-test

### Comparison of liver function indexes before and after DEB-TACE and cTACE

Of the 23 patients who received hepatectomy after TACE as a down-staging therapy (DEB-TACE: 15, cTACE: 8), we collected the liver function indexes and the tumor markers AFP data before and after DEB-TACE at 1 month in these patients. When compared to the liver function indexes before and after TACE in each group or between the two group, no difference was found in liver function indexes, including ALB, TP, TBIL, TBA, ALT, and AST (all *p* > 0.05, Table 5), while the AFP level in the DEB-TACE group was significantly lower than that in the cTACE group (*p* = 0.003, Table 5).

**Table 5:** Comparison of liver function indexes before and after DEB-TACE and cTACE

Liver function indexes	DEB-TACE group (*N* = 15)		cTACE group (*N* = 8)		DEB-TACE vs cTACE	
	Before TACE	After TACE 1 month	Before TACE	After TACE 1 month	*p-value*^*a*^	*p-value*^*b*^
ALB (g/L)	42.4 ± 3.75	39.8 ± 4.24	41.9 ± 4.32	38.9 ± 4.15	0.775	0.630
TP (g/L)	70.5± 5.62	67.4 ± 3.69	71.7 ± 4.21	69.4 ± 3.78	0.603	0.233
TBIL (μmol/L)	12.51 ± 6.28	19.67 ± 8.54	14.29 ± 4.36	16.44 ± 3.54	0.484	0.322
TBA (μmol/L)	7.3 ± 3.24	8.1 ± 2.25	8.6 ± 4.63	8.9 ± 3.47	0.439	0.509
ALT (U/L)	41.4 ± 18.21	46.8 ± 15.37	39.14 ± 19.21	40.95 ± 20.88	0.784	0.451
AST (U/L)	43.57 ± 25.33	48.63 ± 16.34	45.27 ± 30.22	46.5 ± 18.25	0.887	0.778
AFP (ng/mL)	1257.4 ± 631.8	268.8 ± 96.5	1182 ± 531.2	421.3 ± 113.2	0.777	0.003^*^

^a^, comparison between DEB-TACE and cTACE before TACE by using *t*-test. ^b^, comparison between DEB-TACE and cTACE at 1 month by using *t*-test

*p < 0.05

### RFS and OS

**Fig. 1:**
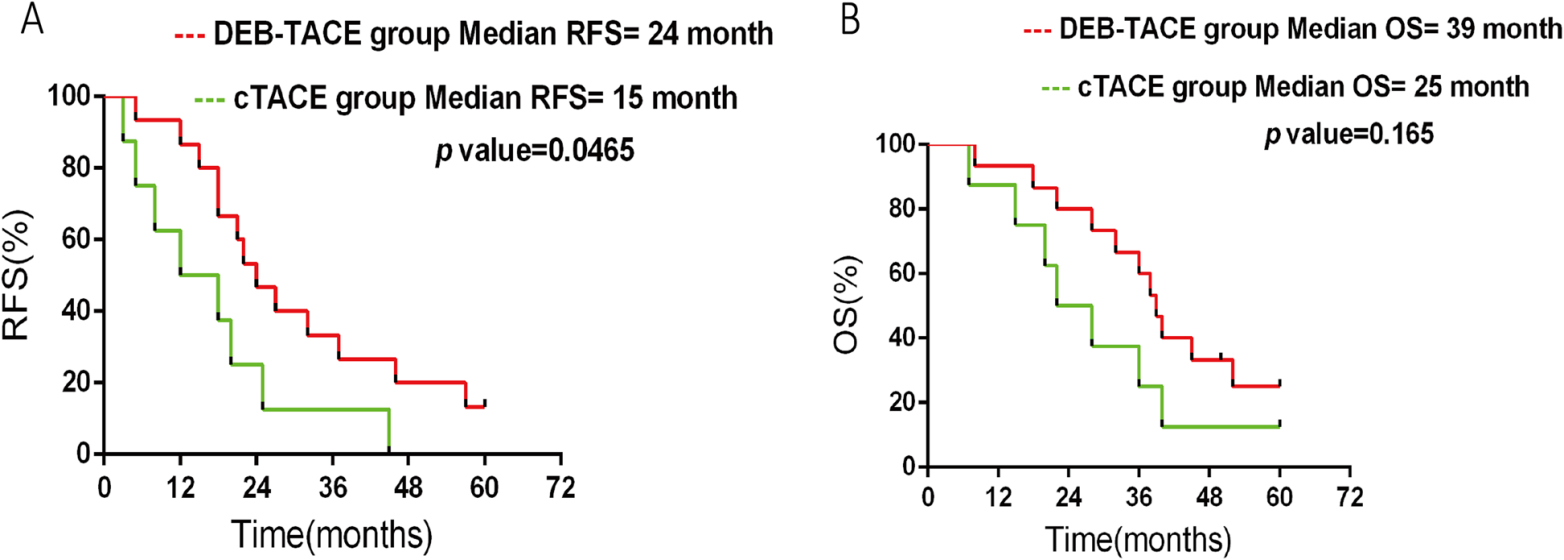
Survival. **A** The patients’ RFS of the DEB-TACE group and cTACE group analyzed by Kaplan–Meier survival curve. **B** The patients’ OS of the DEB-TACE group and cTACE group analyzed by Kaplan–Meier survival curve

Of the 23 patients who received hepatectomy after TACE as a down-staging therapy (DEB-TACE: 15, cTACE: 8), for survival, the median RFS was 26.0 months in DEB-TACE group and 15 months in cTACE group; there was significant difference between the two groups (*p* = 0.0465, Fig. 1A). As to OS, the median OS in DEB-TACE group was higher than that in cTACE group, but there was no significant difference between the two group in terms of OS (*p* = 0.165, Fig. 1B).

### Adverse events after DEB-TACE and cTACE

Of the 23 patients who received hepatectomy after TACE as a down-staging therapy (DEB-TACE:15, cTACE:8), when compared the adverse events after TACE between the two group, no difference was found in complications, including pain, fever, nausea/vomiting, transient liver injury, liver abscess, ascites, and myelosuppression (all *p* > 0.05, Table 6).

**Table 6:** Adverse events after DEB-TACE and cTACE

Complications	DEB-TACE (*N* = 15)	cTACE (*N* = 8)	*p-value*
Pain, no. (%)	6 (40%)	4 (50%)	0.685
Fever, no. (%)	7 (46.7%)	3 (37.5%)	1.000
Nausea/vomiting, no. (%)	5 (33.3%)	4 (50%)	0.657
Transient liver injury, no. (%)	7 (46.7%)	5 (62.5%)	0.710
Liver abscess, no. (%)	0 (0%)	0 (0%)	1.000
Ascites, no. (%)	2 (13.3%)	2 (25%)	0.589
Myelosuppression, no. (%)	1 (6.7%)	1 (12.5%)	1.000

### Typical case report of DEB-TACE for down-staging in unresectable liver cancer

**Fig. 2:**
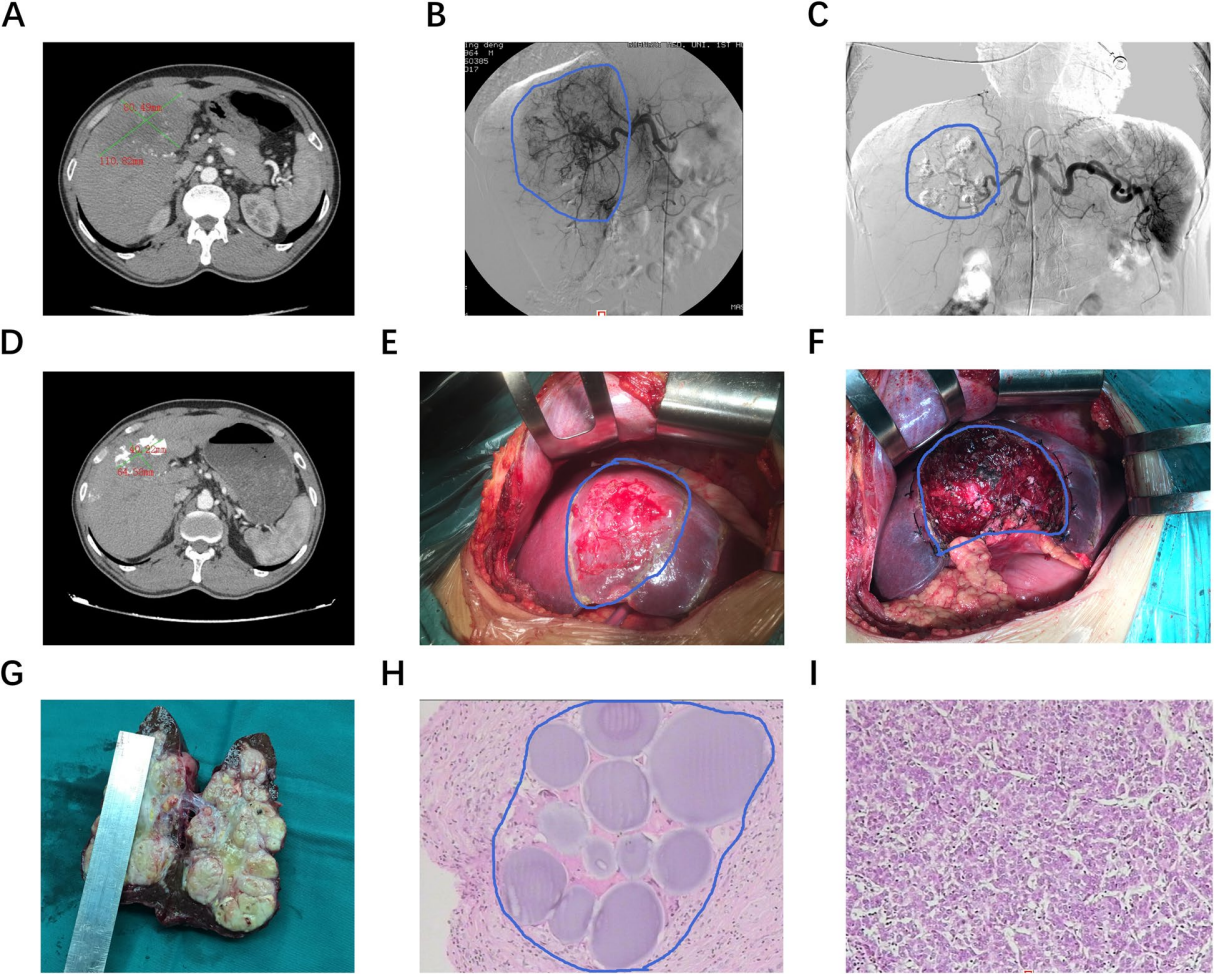
Typical case report. **A** Representative images of tumor by abdominal computed tomography scan before DEB-TACE treatment. **B** Representative images of DEB-TACE treatment. **C** Representative images of hepatic arteriography during DEB-TACE. **D** Representative images of tumor abdominal computed tomography scan after DEB-TACE treatment. **E**–**F** Representative images of tumor during resection. **G** Representative images of resected tumor. **H**–**I** Representative images of tumor pathological examination

Mr. Huang, a 56-year-old man, was first diagnosed as HCC. Before DEB-TACE treatment, abdominal computed tomography (CT) scan showed that tumor size was 80.49 mm × 110.82 mm (Fig. 2A). After identifying the tumor supply artery by hepatic arteriography, DEB-TACE treatment was performed (Fig. 2B). After the operation, hepatic arteriography was carried out immediately, which disclosed that the tumor was reduced and the tumor blood supply arteries were almost stagnant (Fig. 2C). Before the surgery, abdominal CT scan showed that tumor size was 40.22 mm × 64.38 mm, which diameter was reduced nearly 5cm (Fig. 2D). After resection (Fig. 2 E, F), the pathological examination was performed (Fig. 2G), which discovered drug-loaded microspheres (Fig. 2H). This patient was pathologically confirmed HCC with a trend of differentiation of bile duct cell carcinoma (Fig. 2I).

## Discussion

DEB-TACE is a new type of embolization material involving the injection of a chemotherapeutic agent selectively into the feeding arteries of the tumor to not only potentially obtain higher intratumor drug concentrations and maintain lower plasma drug concentrations compared with cTACE but also block the blood vessel effectively causing infarction and necrosis [9]. Accumulating evidence reveals that the 1-month CR rate ranges from 19.2 to 70.6% in tumors, and ORR ranges from 56.3 to 100% in tumors [13, 14]. In line with these previous data, we discovered that the 1-month CR rate and ORR in patients were 20.8% and 70.8% in DEB-TACE group while in cTACE group were 6.25% and 34.4%, respectively, which indicated that compared with cTACE, DEB-TACE can obtain higher CR rate and ORR rate in patients.

Based on the management guidelines published by the American Association for the Study of Liver Diseases, curative treatments have been only recommended to liver cancer patients who are with a single nodule < 5 cm in diameter, three or fewer nodules < 3 cm in diameter, or meet the Milan criteria [15, 16]. Although DEB-TACE is related to promising efficacy and low toxicity in unresectable liver cancer patients, which could effectively delay tumor progression or prevent recurrence during short term (within 6 months), it is still less effective over longer periods and could not achieve response rates and cure the tumor comparable to curative therapy [17]. There is therefore a requirement with additional and effective treatment strategies for unresectable liver cancer patients, including the optimization of DEB-TACE and its combination with other treatment modalities [18, 19]. TACE has been regarded as preoperative neoadjuvant chemotherapy to improve outcomes in patients with resectable hepatocellular carcinoma (HCC), which also is referred to as “bridging” therapy before liver transplantation for HCC [20, 21]. However, limited information about the down-staging effect of DEB-TACE in unresectable liver cancer patients. One previous study enrolling 30 unresectable liver cancer patients listed to liver transplantation discloses that after 3 times DEB-TACE successfully, 76.7% patients are down-staged to meet the University of California, San Francisco (UCSF) criteria, and 53.3% patients are down-staged to meet Milan criteria; meanwhile, there are 13 patients had been given liver transplantation successfully [22, 23].

As the advantage of DEB-TACE discussed above, we hypothesized DEB-TACE can be used as a neoadjuvant treatment for unresectable liver cancer patients, which might have down-staging effects for those patients and made them receive curative treatment after DEB-TACE treatment, whereas there was no evidence about whether DEB-TACE as down-staging therapy in unresectable liver cancer patients listed to receive surgery. Thus, we performed this study and discovered that DEB-TACE decreased tumor diameter in unresectable liver cancer patients; meanwhile, we found that DEB-TACE promoted the growth of residual liver volume in unresectable liver cancer patients. The possible explanations were that DEB-TACE is not only related to a high and sustained chemotherapy drug concentration but also benefited for embolization of the small artery supplying tumor, which guaranteed the efficacy of DEB-TACE treatment on killing tumor cells and blocking tumor blood supply to promote tumor necrosis and inhibit tumor growth, thereby contributing to down-staging effect in unresectable liver cancer patients.

More importantly, we discovered that 18.8% patients could receive resection after DEB-TACE, suggesting that the rate for unresectable patients who received resection was higher than cTACE. For survival, the median RFS was 26.0 months in DEB-TACE group and 15 months in cTACE group; there was significant difference between the two group. As to OS, the median OS in DEB-TACE group was higher than that in cTACE group. These results were in line with a previous Chinese study disclosing the percentage of 1-year accumulating RFS of 92.3% and the percentage of 1-year accumulating OS of 100.0% [23].

Like each invasive treatment, DEB-TACE undertakes the risk of AEs, including abdominal pain, fever, vomiting, and increased blood pressure [24]. In this study, we also discovered that no difference was found in complications, including pain, fever, nausea/vomiting, transient liver injury, liver abscess, ascites, and myelosuppression between the DEB-TACE group and cTACE group. These results indicated that there were no serious complications occurring in these unresectable liver cancer patients after DEB-TACE, which showed the advantage of DEB-TACE with low systemic toxicity and good safety.

There were still several limitations in this study. The main limitation was a relatively small sample size, which might lead to poor statistical power. Further validation is necessary in a larger sample size. In addition, the relatively short follow-up duration existed in this study; hence, long-term efficacy and safety were not able to be evaluated. Furthermore, all unresectable liver cancer patients were from one hospital; there might be selected bias. Further study with more patients from a multicenter is necessary.

In summary, DEB-TACE might be effective and safe as a down-staging therapy in unresectable liver cancer patients.

## Data Availability

Datasets used and/or analyzed during the current study are available from the corresponding author on reasonable request.

## Abbreviations

DEB-TACE: Drug-eluting beads transarterial chemoembolization
cTACE: Conventional transarterial chemoembolization
CNLC: China liver cancer staging
RFS: Relapse-free survival
OS: Overall survival
CR: Complete response
ORR: Objective response rate
PHC: Primary hepatocellular carcinoma
HCC: Hepatocellular carcinoma
PR: Partial response
CT: Computed tomography
AFP: Alpha-fetoprotein
ALB: Albumin
TP: Total protein
TBIL: Total bilirubin
TBA: Total bile acid
ALT: Alanine aminotransferase
AST: Aspartate aminotransferase
MRI: Magnetic resonance imaging

## References

[1] Mohammadian M, Allah Bakeshei K, Mohammadian-Hafshejani A. International epidemiology of liver cancer: geographical distribution, secular trends and predicting the future[J]. J Prev Med Hyg. 2020;61(2):E259–E289. 10.15167/2421-4248/jpmh2020.61.2.1244. 10.15167/2421-4248/jpmh2020.61.2.1244 PMC7419131 32803012

[2] Forner A, Llovet JM, Bruix J (2012). Hepatocellular carcinoma [J]. Lancet.

[3] Alejandro F, Mar AR, Jordi B (2018). Hepatocellular carcinoma [J]. Lancet.

[4] Lencioni R, Crocetti L (2013). Image-guided ablation for hepatocellular carcinoma [J]. Recent Results Cancer Res.

[5] Breen DJ, Lencioni R (2015). Image-guided ablation of primary liver and renal tumours [J]. Nat Rev Clin Oncol.

[6] Norihiro I, Masatoshi (2014). Transarterial chemoembolization for hepatocellular carcinoma: a review of techniques [J]. World J Hepatol.

[7] Li L, Cheng N, Huang X, Weng X, Jiao Y, Liu J, Guo W. Efficacy and safety of endovascular brachytherapy combined with transarterial chemoembolization for the treatment of hepatocellular carcinoma patients with type III or IV portal vein tumor thrombosis[J]. World J Surg Oncol. 2022;20(1):30. 10.1186/s12957-022-02495-4. 10.1186/s12957-022-02495-4 PMC8808970 35109883

[8] Ni JY, Xu LF, Wang WD (2014). Conventional transarterial chemoembolization vs microsphere embolization in hepatocellular carcinoma: a meta-analysis [J]. World J Gastroenterol.

[9] Chen P, Yuan P, Chen B, Sun J, Shen H, Qian Y. Evaluation of drug-eluting beads versus conventional transcatheter arterial chemoembolization in patients with unresectable hepatocellular carcinoma: a systematic review and meta-analysis[J]. Clin Res Hepatol Gastroenterol. 2017;41(1):75–85. 10.1016/j.clinre.2016.05.013. 10.1016/j.clinre.2016.05.013 27350573

[10] Zhou GH, Han J, Sun JH (2018). Efficacy and safety profile of drug-eluting beads transarterial chemoembolization by CalliSpheres beads in Chinese hepatocellular carcinoma patients [J]. BMC Cancer.

[11] Xin Z, Xiao (2019). An investigation of efficacy, safety, and prognostic factors of drug-eluting beads-transarterial chemoembolization operation with CalliSpheres? Microspheres in treating Chinese hepatocellular carcinoma patients [J]. J Clin Lab Anal.

[12] Wu B, Zhou J, Ling G (2018). CalliSpheres drug-eluting beads versus Lipiodol transarterial chemoembolization in the treatment of hepatocellular carcinoma: a short-term efficacy and safety study [J]. World J Surg Oncol.

[13] Nani, Trevisani, Gasparini (2014). Randomised controlled trial of doxorubicin-eluting beads vs conventional chemoembolisation for hepatocellular carcinoma [J]. The. Br J Cancer.

[14] Sacco R, Bargellini I, Bertini M (2011). Conventional versus doxorubicin-eluting bead transarterial chemoembolization for hepatocellular carcinoma [J]. J Vasc Int Radiol.

[15] Bruix J, Sherman M (2011). Management of hepatocellular carcinoma: an update [J]. Hepatology.

[16] Mazzaferro V, Regalia E, Doci R (1996). Liver transplantation for the treatment of small hepatocellular carcinomas in patients with cirrhosis [J]. N Engl J Med.

[17] Jin-Woo L, Hye JJ, Jin YJ (2015). Transarterial chemoembolization for hepatocellular carcinoma: an evidence-based review of its place in therapy [J]. J Hepatocell Carcinoma.

[18] Cheng Z, He L, Guo Y (2020). The combination therapy of transarterial chemoembolisation and sorafenib is the preferred palliative treatment for advanced hepatocellular carcinoma patients: a meta-analysis [J]. World J Surg Oncol.

[19] Jiang C, Cheng G, Liao M, Huang J. Individual or combined transcatheter arterial chemoembolization and radiofrequency ablation for hepatocellular carcinoma: a time-to-event meta-analysis[J]. World J Surg Oncol. 2021;19(1):81. 10.1186/s12957-021-02188-4. 10.1186/s12957-021-02188-4 PMC7980330 33741001

[20] Nishikawa H, Arimoto A, Wakasa T, Kita R, Kimura T, Osaki Y. Effect of transcatheter arterial chemoembolization prior to surgical resection for hepatocellular carcinoma[J]. Int J Oncol. 2013;42(1):151–60. 10.3892/ijo.2012.1711. Epub 2012 Nov 21. 10.3892/ijo.2012.1711 23174998

[21] Fujiki M, Aucejo F, Kim R (2011). General overview of neo-adjuvant therapy for hepatocellular carcinoma before liver transplantation: necessity or option? [J]. Liver Int.

[22] Abdelfattah MR, El-Siesy H, Al-Manea H (2018). Liver transplantation for hepatocellular carcinoma within the Milan criteria versus the University of California San Francisco criteria: a comparative study [J]. Eur J Gastroenterol Hepatol.

[23] Cai L, Li H, Guo J (2021). Drug-eluting bead transarterial chemoembolization is an effective downstaging option for subsequent radical treatments in patients with hepatocellular carcinoma: a cohort study [J]. Gastroentérologie Clinique et Biologique..

[24] Zou JH, Zhang L, Ren ZG (2016). Efficacy and safety of cTACE versus DEB-TACE in patients with hepatocellular carcinoma: a meta-analysis [J]. J Dig Dis..

